# Angiogenesis-related lncRNAs predict the prognosis signature of stomach adenocarcinoma

**DOI:** 10.1186/s12885-021-08987-y

**Published:** 2021-12-07

**Authors:** Chen Han, Cong Zhang, Huixia Wang, Kexin Li, Lianmei Zhao

**Affiliations:** Research Center, The Fourth Hospital of Hebei Medical University, 050011 Shijiazhuang, China

**Keywords:** Stomach adenocarcinoma, Angiogenesis, Long noncoding RNA, Prognosis, Signature

## Abstract

**Background:**

Stomach adenocarcinoma (STAD), which accounts for approximately 95% of gastric cancer types, is a malignancy cancer with high morbidity and mortality. Tumor angiogenesis plays important roles in the progression and pathogenesis of STAD, in which long noncoding RNAs (lncRNAs) have been verified to be crucial for angiogenesis. Our study sought to construct a prognostic signature of angiogenesis-related lncRNAs (ARLncs) to accurately predict the survival time of STAD.

**Methods:**

The RNA-sequencing dataset and corresponding clinical data of STAD were acquired from The Cancer Genome Atlas (TCGA). ARLnc sets were obtained from the Ensemble genome database and Molecular Signatures Database (MSigDB, Angiogenesis M14493, INTegrin pathway M160). A ARLnc-related prognostic signature was then constructed via univariate Cox and multivariate Cox regression analysis in the training cohort. Survival analysis and Cox regression were performed to assess the performance of the prognostic signature between low- and high-risk groups, which was validated in the validation cohort. Furthermore, a nomogram that combined the clinical pathological characteristics and risk score conducted to predict the overall survival (OS) of STAD. In addition, ARLnc-mRNA coexpression pairs were constructed with Pearson’s correlation analysis and visualized to infer the functional annotation of the ARLncs by gene ontology (GO) and Kyoto Encyclopedia of Genes and Genomes (KEGG) pathway analysis. The expression of four ARLncs in STAD and their correlation with the angiogenesis markers, CD34 and CD105, were also validated by RT–qPCR in a clinical cohort.

**Results:**

A prognostic prediction signature including four ARLncs (PVT1, LINC01315, AC245041.1, and AC037198.1) was identified and constructed. The OS of patients in the high-risk group was significantly lower than that of patients in the low-risk group (*p* < 0.001). The values of the time-dependent area under the curve (AUC) for the ARLnc signature for 1-, 3-, and 5- year OS were 0.683, 0.739, and 0.618 in the training cohort and 0.671, 0.646, and 0.680 in the validation cohort, respectively. Univariate and multivariate Cox regression analyses indicated that the ARLnc signature was an independent prognostic factor for STAD patients (*p* < 0.001). Furthermore, the nomogram and calibration curve showed accurate prediction of the survival time based on the risk score. In addition, 262 mRNAs were screened for coexpression with four ARLncs, and GO analysis showed that mRNAs were mainly involved in biological processes, including angiogenesis, cell adhesion, wound healing, and extracellular matrix organization. Furthermore, correlation analysis showed that there was a positive correlation between risk score and the expression of the angiogenesis markers, CD34 and CD105, in TCGA datasets and our clinical sample cohort.

**Conclusion:**

Our study constructed a prognostic signature consisting of four ARLnc genes, which was closely related to the survival of STAD patients, showing high efficacy of the prognostic signature. Thus, the present study provided a novel biomarker and promising therapeutic strategy for patients with STAD.

**Supplementary Information:**

The online version contains supplementary material available at 10.1186/s12885-021-08987-y.

## Background

Gastric cancer (GC) is the fifth most common malignant tumor and the fourth leading cause of cancer-related deaths in the world [1]. Stomach adenocarcinoma (STAD) is the most common histological type and accounts for 90% of gastric cancer. As onset is insidious, most patients are already in the advanced stage of cancer when they are first diagnosed with STAD. Although various traditional treatments including surgery, radiotherapy, chemotherapy, and target drugs have been used for early interventions [2, 3], the efficacy is still limited for advanced gastric cancer, and the 5-year survival rate of advanced gastric cancer is less than 10% [4]. Identification of new and effective potential diagnostic biomarkers as well as development of novel treatments are urgently needed.

Tumor angiogenesis is the process of new blood vessel formation from preexisting blood vessels, and it is responsible for adequate supplies of oxygen and nutrients for tumors as well as a gateway for tumor cells to enter the blood stream and metastasize [5]. The shift in the balance of pro- and anti- angiogenic factors is termed the “angiogenic switch” [6]. As the tumor progress beyond microscopic size, hypoxia induces the production of various pro-angiogenic factors, such as growth factors, chemokines, extracellular matrix (ECM) components and integrins, which leads to enhanced, rapid, and chaotic blood vessel formation, ultimately leading to the “angiogenic switch” [7].

LncRNAs are defined as nonprotein coding transcripts of more than 200 nucleotides that are transcribed by the enzyme RNA polymerase II or RNA polymerase III, and they do not encode proteins (although small peptides are encoded by some lncRNAs) [8]. LncRNAs have been proposed to perform diverse functions involved in the classic hallmarks of cancer, including accelerated proliferation, immune evasion, induction of angiogenesis, resistance to cell death, and regional or distant metastasis [9]. Abnormally expressed lncRNAs have been found in STAD, and deregulated lncRNAs contribute to hallmarks of cancer, ranging from the intrinsic capacity of proliferation and survival to the tumor microenvironment [10]. In addition to their possible role in cancer biology, lncRNAs have emerged as a new class of promising biomarkers for diagnosis and prognosis. Compared with mRNA expression, lncRNA expression is more specific in different tissues, which may provide more information for identifying specific biomarkers for tumors [11]. Growing evidence on the influence of angiogenesis-related lncRNAs (ARLncs) suggests that these transcripts are potential predictive, diagnostic biomarkers or therapeutic targets for antiangiogenesis treatment. Lei et al. identified a five angiogenesis-related lncRNA signature (LINC01138, LINC00942, AL031985.3, AC015908.3 and USP46-AS) as an independent prognostic biomarker for predicting hepatocellular carcinoma patient survival [12]. However, there has been no signature to systematically assess ARLncs in STAD, and it remains unclear whether ARLncs can be used to predict the survival of STAD patients. In the present study, we first established a prognostic signature consisting of four ARLncs (PVT1, LINC01315, AC245041.1, and AC037198.1) for STAD patients, which was verified as a key prognostic predictor and may serve as a potential therapeutic target for STAD.

## Methods

### Data download and pretreatment

The transcriptome RNA-sequencing data (HTSeq-fragments per kilobase million, HTSeq-FPKM) and clinical information of STAD patients were downloaded from The Cancer Genome Atlas (TCGA) data portal (http://portal.gdc.cancer.gov/), which contained data from 375 STAD and 32 normal stomach tissues (updated in November 11, 2019). RNA-Seq expression level read counts produced by HT-Seq were normalized using FPKM method. Clinical information (including age, gender, grade, survival time, survival status, and TNM stage) was extracted and integrated using Perl (http://www.perl.org/get.html). The criteria for the inclusion of STAD samples were complete lncRNA expression value and clinical information. Patients with a survival time < 30 days (*n* = 28) were excluded to eliminate noncancer-related deaths. Furthermore, patient samples with missing clinical information (stage, *n* = 11; T stage, *n* = 15; N stage, *n* = 2) were excluded from the study. The annotation of RNA was obtained from Ensemble Human GRCh38.p13 database (http://www.ensembl.org/). mRNAs and lncRNAs were extracted with Perl and further used for the following data analysis.

### Angiogenesis- related lncRNA (ARLnc) acquisition

The Molecular Signatures Database v7.4 (Angiogenesis M14493, INTegrin pathway M160, http://www.gsea-msigdb.org/gsea/msigdb/) was used to specify angiogenesis-related genes participating in the angiogenesis process. Pearson correlation analysis was conducted by the “limma” package in R to calculate the correlation coefficient between lncRNAs and angiogenesis-related genes. According to the analysis, lncRNAs with |cor| > 0.4 and *p* < 0.05 were defined as angiogenesis-related lncRNAs and screened for follow- up study.

### Prognostic signature constructed with ARLncs

To confirm the potential prognosis-related ARLncs, Kaplan–Meier survival analysis and univariate Cox proportional risk regression analysis were performed to evaluate the association between the expression of the screened ARLncs and OS in the training dataset. Next, the ARLncs with *p* < 0.05 were included in multivariate Cox regression analysis, and an ARLnc-based prognostic risk model was established based on the linear combination of the regression coefficients and their expression levels obtained from the multivariate Cox regression model. The detailed formula was follows: risk score = [Expression level of PVT1 × (− 0.41723)] + [Expression level of AC245041.1 × (0.027081)] + [Expression level of LINC01315 × (− 0.19828)] + [Expression level of AC037198.1 × (0.245153)]. The calculated median was then used as the tipping point for the risk score to classify STAD patients into a high-risk group (with a score above the median) and a low-risk group (with a score below the median).

To analyze whether the survival time of the patients in both risk groups was significantly different, a Kaplan–Meier survival curve was used and visualized by the “survival” package in R. To verify the prognostic accuracy of the ARLnc-related prediction signature, receiver operating characteristic (ROC) curve and the area under the curve (AUC) were calculated to evaluate the significance of the survival difference between the high- and low-risk groups via the “survival ROC” and the “ggplot2” packages in R.

### Data processing

Principal component analysis (PCA) was performed with “R” to obtain the expression patterns of optimal ARLncs in the low-risk and high-risk groups. Moreover, gene set enrichment analysis (GSEA) was also performed for each risk group (http://www.gsea-msigdb.org/gsea/index.jsp). Statistical significance was expressed by the normalized enrichment score (NES), and gene sets with a false discovery rate (FDR) value < 0.01 were considered to be significantly enriched.

### Construction of the nomogram survival model

To assess the probabilities of OS at 1, 3, and 5 years for STAD patients, a nomogram consisting of age, gender, stage and risk score was constructed. In addition, a calibration curve was used to assess the degree to which the actual results were consistent with the predicted results of the nomogram. The concordance index (c-index) and decision curve analysis (DCA) were used to estimate the predictive capability and reliability of the nomogram by using the “rms” package in R software.

### ARLnc-mRNA Coexpression network

Based on the expression levels of ARLncs and mRNAs, Pearson’s correlation coefficient was used to evaluate the correlation between ARLnc and mRNA based on the expression value. The correlation coefficient > 0.45 and FDR < 0.01 were selected. The network was visualized using Cytoscape software (version 3.7.2).

### Functional annotation of mRNA interacting with ARLnc

The functions of coexpressed mRNAs were analyzed by Gene Ontology (GO), Kyoto Encyclopedia of Genes and Genomes (KEGG) pathway analyses using the Database for Annotation, Visualization and Integrated Discovery (DAVID) (http://david.abcc.ncifcrf.gov/tools.jsp). The enrichment and functions of genes inbiological process (BP), cellular component (CC), and molecular function (MF) were analyzed. *P* < 0.05 was selected as the cutoff criterion for GO functional and KEGG pathway enrichment analyses.

### Clinical sample and reverse transcription-quantitative PCR

Tumor tissues and paired adjacent normal tissues from 30 patients with pathologically and clinically diagnosed STAD were prospectively collected from The Fourth Affiliated Hospital of Hebei Medical University from January 2019 to December 2020. The study was approved by the Ethics Committee of The Fourth Affiliated Hospital of Hebei Medical University (Approval Number: 2019054), and written prior informed consent and approval were obtained from all patients. Total RNA was isolated from tissues using TRIzol reagent (Invitrogen). RNA (2 μg) was reversely transcribed into cDNA using a Superscript First-strand Synthesis system (Promega, USA) according to the manufacturer’s instructions. Quantitative RT–PCR was performed using SYBR Premix Ex Tag II (Promega, USA.) on an ABI7500 instrument (Applied Biosystems, USA). The expression levels of ARLnc (PVT1, LINC01315, AC245041.1, and AC037198.1) and angiogenesis-related markers (CD34 and CD105) were normalized to GAPDH and calculated by the 2^−ΔΔCt^ method. Each sample was performed in triplicate. The sequences of the primers utilized in this study were shown in Supplementary Table 3.

### Statistical analysis

All data were processed using the PERL programming language (version 5.30.2), and statistical analyses and graphics were performed using R software (version 3.6.1, Institute for Statistics and Mathematics, http://www.r-project.org) and SPSS v23.0. Chi-square test and Student’s test were used to evaluate qualitative and quantitative variables, respectively. *p* < 0.05 was considered statistically significant.

## Results

### Identification of ARLncs in TCGA STAD dataset

**Fig. 1:**
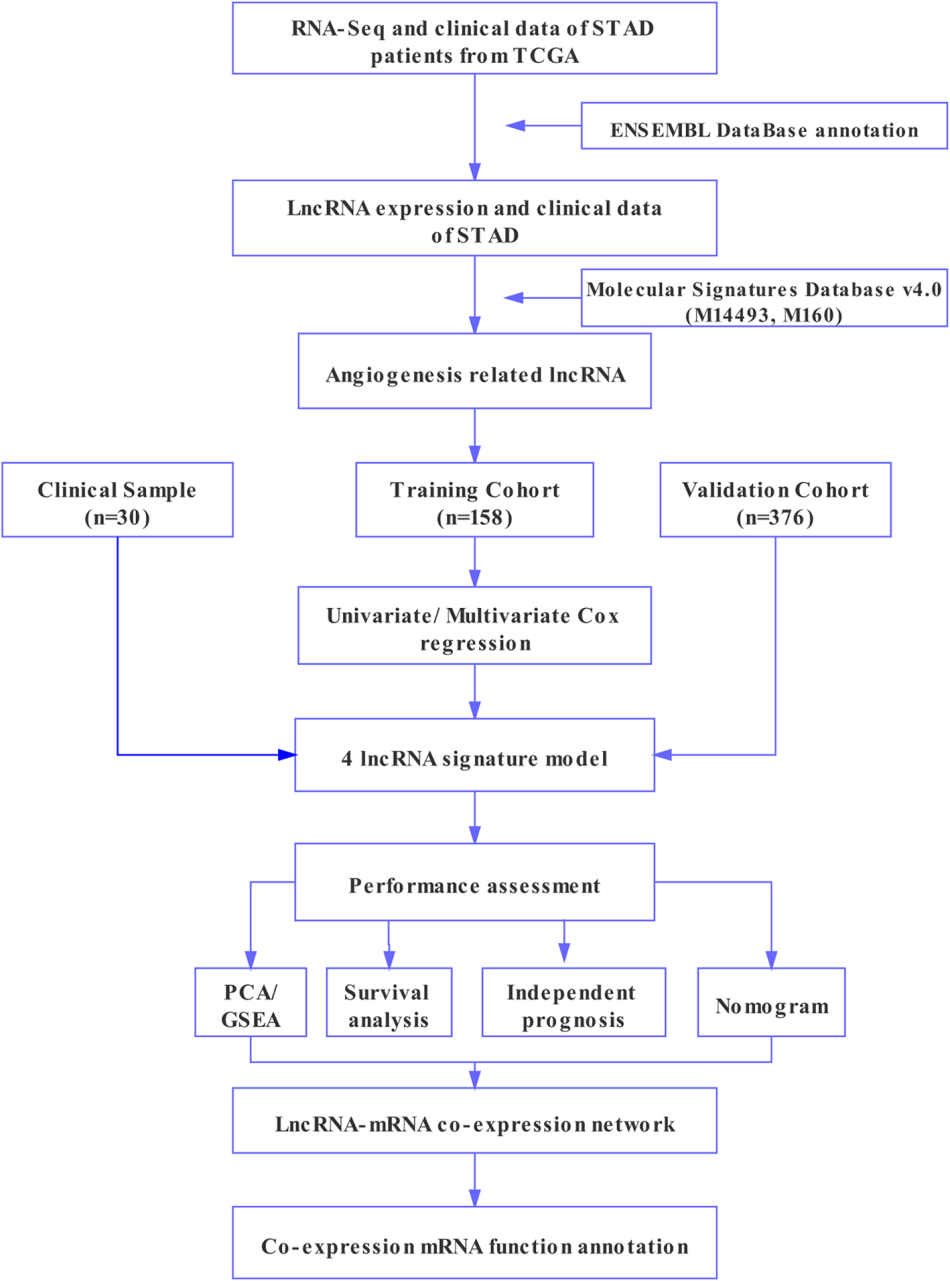
The analysis and design workflow for the ARLnc signature of STAD

A complete workflow for our research process was depicted in Fig. 1. We identified a total of 14,143 lncRNAs in the STAD dataset, which was extracted from TCGA database. Meanwhile, 119 angiogenesis-related genes were extracted from the Molecular Signatures Database v4.0 (Table S1). Finally, according to the screening criteria of |cor| > 0.4 and *p* < 0.05 in Pearson correlation analysis, 329 lncRNAs were defined as ARLncs and screened for follow-up study (Table S2).

### Clinical characteristics of STAD patients

After the exclusion of patients with survival time < 30 days and with missing survival information, 319 STAD patients were involved in the construction and validation of the ARLnc signature. Of these patients, 158 were assigned to a training cohort utilizing the bootstrapping approach (1000 resamplings) for a more precise representation of the population, and the entire dataset was used as the validation cohort. The clinical characteristics of the 319 STAD patients were shown in Table 1. There were no significant differences in the gender (*p* = 0.840), age (*p* = 0.623), TNM stage (*p* = 0.697), or survival status (*p* = 0.610) distribution between the training cohort and validation cohort.

**Table 1:** The clinical features of STAD patients in training cohort and validation cohort

Character	Training cohort	Entire cohort	*p* value
	*n* = 158	*n* = 319	
**Age(year)**			
≥ 65	94(59.50%)	181(56.74%)	0.623
<65	64(40.50%)	138(43.26%)	
**Gender**			
Female	59(37.34%)	115(36.05%)	0.840
Male	99(62.66%)	204(63.95%)	
**TNM Stage**			
I-II	71(44.94%)	150(47.02%)	0.697
III-IV	87(55.06%)	169(52.98%)	
**T stage**			
T1-T2	41(25.95%)	81(25.39%)	0.911
T3-T4	117(74.05%)	238(74.61%)	
**M stage**			
M1	8(5.06%)	21(6.58%)	0.684
M0-Mx	150(94.94%)	298(93.42%)	
**N stage**			
N0-N1	87(55.06%)	182(57.05%)	0.696
N2-N3	71(44.94%)	137(42.95%)	
**Futime**			
**Survival status**			
Alive	106(67.09%)	205(64.26%)	0.610
Dead	52(32.91%)	114(35.74%)	

### Identification and validation of the ARLnc prognostic signature in the training and validation cohorts

**Fig. 2:**
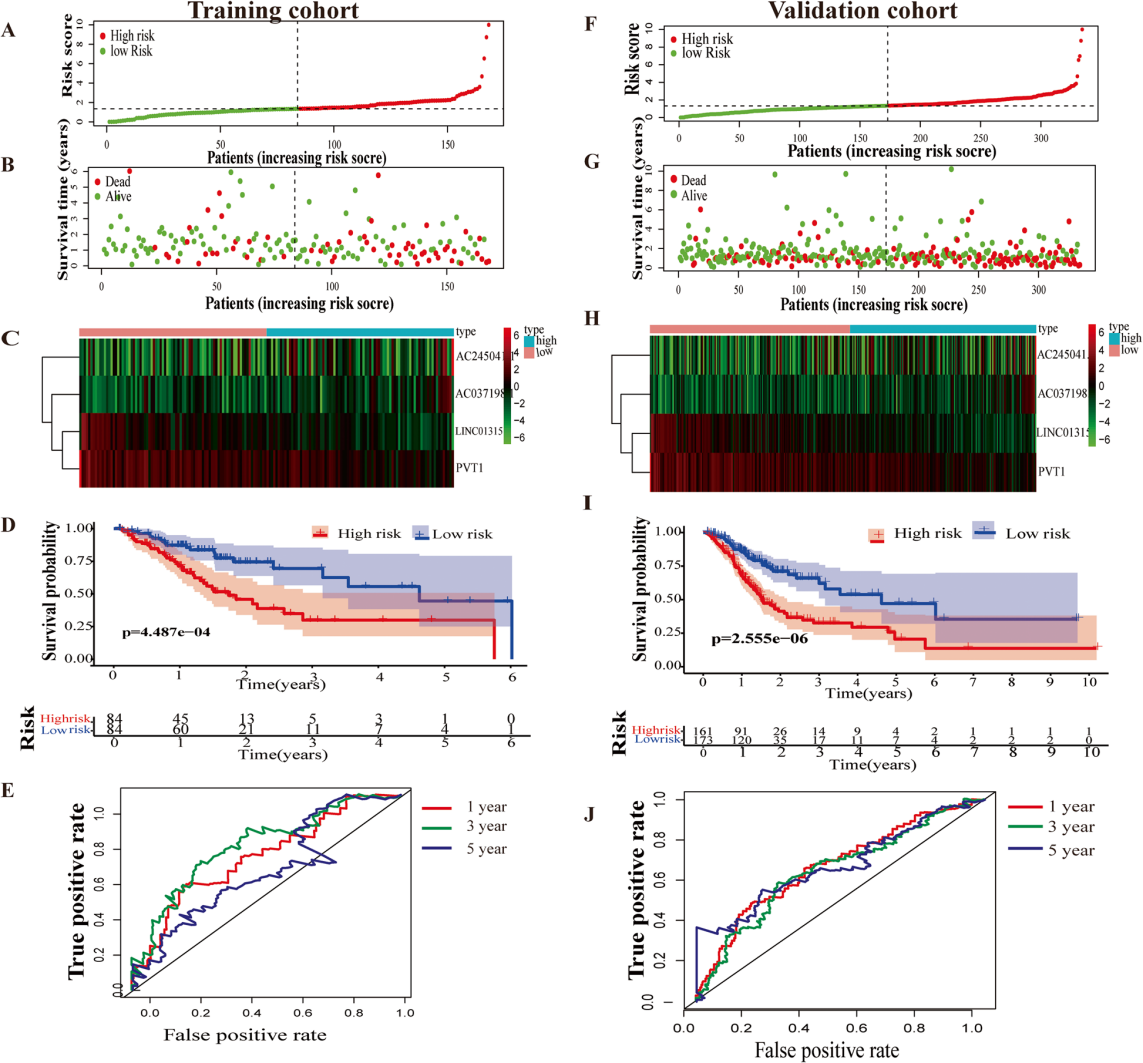
Construction and validation of the ARLnc predictive signature in STAD patients. Distribution of risk score, survival status, expression of four ARLncs in the high- and low- risk groups of STAD patients in the training cohort (**A-C**), and in the validation cohort; (**F-H**). Survival difference between high- and low-risk groups in the training cohort (**D**) and in the validation cohort (**I**); The AUCs for the ARLnc signature at 1-, 3-, and 5-year OS in the training (**E**) and validation cohort (**J**), respectively

To identify the prognostic value of ARLnc in STAD patients, subsequent univariate and multivariate Cox regression analyses were performed in the training cohort. As shown in Table 2, the signature consisting of four lncRNAs (PVT1, LINC01315, AC245041.1, and AC037198.1) was identified as a potential prognostic biomarker for STAD, and the risk score of each patient in the training cohort was calculated based on the expression and coefficients of the four lncRNAs by multivariate Cox regression. As shown in Fig. 2A-B, the distribution of the risk score and survival duration of gastric cancer patients demonstrated that the risk score was inversely proportional to the survival of patients with STAD, and the patients in the high-risk group had a worse prognosis. In addition, the expression profiles plotted by the risk heatmap of the ARLncs showed that with increasing risk score, the expression levels of AC245041.1 and AC037198.1 were elevated, while the expression levels of PVT1 and LINC01315 were decreased (Fig. 2C). Furthermore, Kaplan–Meier survival curve analysis revealed that patients with high-risk scores had an significantly poorer OS than those with low-risk scores (*p* = 4.487e-06, Fig. 2D). ROC analysis was used to evaluate the predictive accuracy of the prognostic signature, and the results showed that the values of time-dependent AUCs for the ARLnc signature at 1-, 3-, and 5-year OS were 0.683, 0.739, and 0.618 in the training cohort, respectively (Fig. 2E).

**Table 2:** Four ARLncs significantly associated with overall survival in the training cohort

	UntiCox		MultiCox		
Gene Symbol	HR (95%CI)	*P* value	HR (95%CI)	*P* value	Coef
LINC01315	0.637973 (0.461715–0.881518)	0.006442	0.658868 (0.474032–0.915777)	0.013002	−0.41723
PVT1	0.815576 (0.687716–0.967207)	0.019119	0.820139 (0.689334–0.975764)	0.025304	−0.19828
AC037198.1	1.339809 (1.057604–1.697315)	0.015348	1.277817 (1.011582–1.61412)	0.039728	0.245153
AC245041.1	1.03031 (1.012153–1.048792)	0.000996	1.027451 (1.008908–1.046334)	0.003563	0.027081

*HR* Hazard ratio, *95%CI* Upper and lower limits for the hazard ratio, *Coef* Coefficient, *P value* the angiogenesis related lncRNA associated with overall survival of GC

Next, the predictive ability of the ARLnc prognostic signature was further validated in the validation cohort. Consistently, 319 patients were divided into two groups with the same cutoff used in the training cohort (Fig. 2F), and the survival duration of 319 patients was determined (Fig. 2G). The expression profiles plotted by the risk heatmap in the validation cohort showed that compared to the low-risk group, the expression level of risk-type lncRNAs (AC245041.1 and AC037198.1) in the high-risk group was elevated, while the expression level of protective-type lncRNAs (PVT1 and LINC01315) was decreased (Fig. 2H). Kaplan–Meier survival curves indicated that patients with high-risk scores had a significantly poorer OS than those with low-risk scores in the validation cohort (*p* = 2.555e-06, Fig. 2I). Moreover, in the validation cohort, the AUCs for 1-, 3-, and 5-year OS were 0.671, 0.646, and 0.680, respectively, showing the good prognostic prediction of the ARLnc-related gene signature (Fig. 2J).

### The ARLnc signature as an independent prognostic biomarker for STAD

**Fig. 3:**
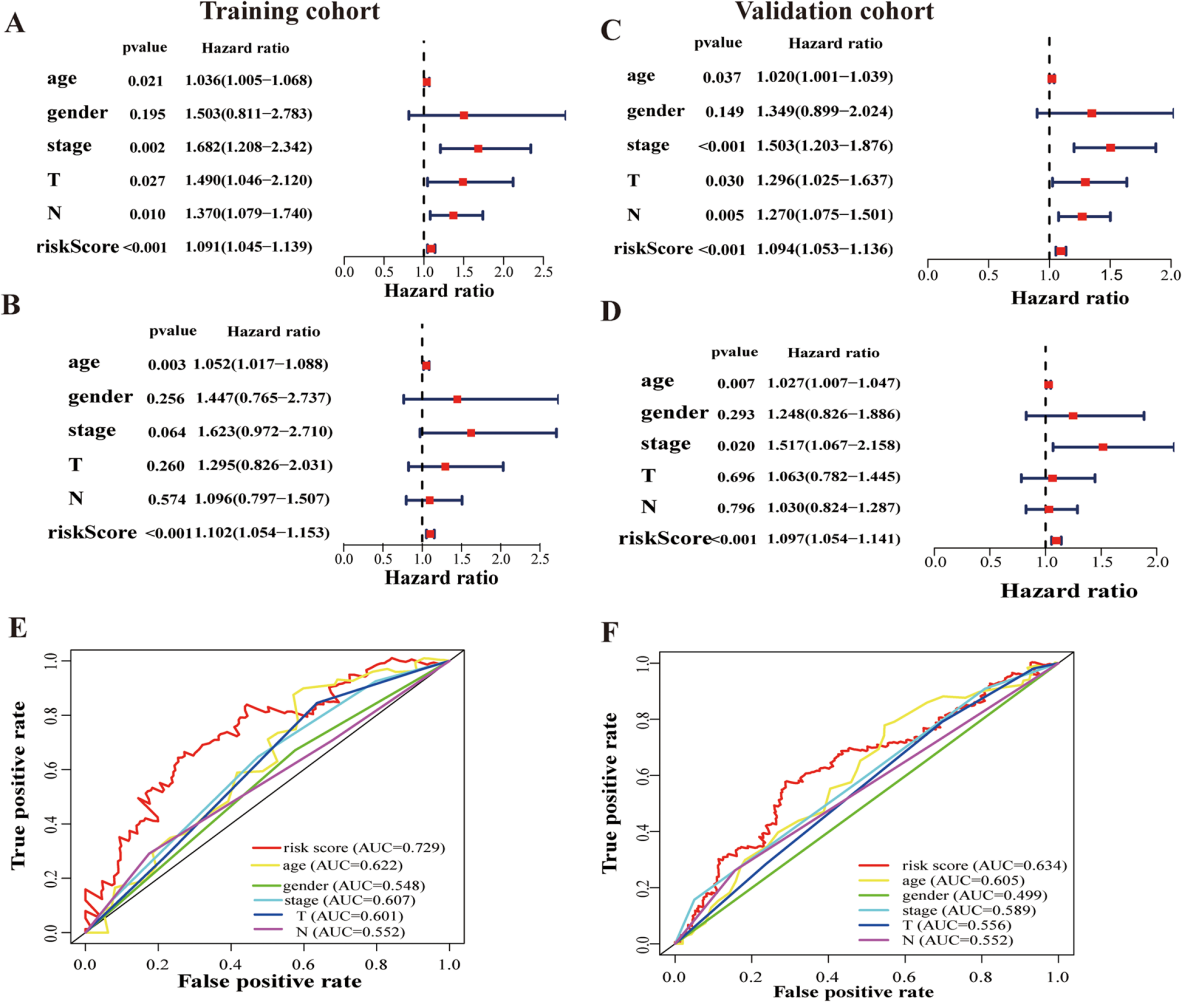
The ARLnc as an independent prognostic factor for the prognosis of STAD patients. **A** Univariate Cox regression and **C** multivariate Cox regression in the training cohort; **B** Univariate Cox regression and **D** multivariate Cox regression in the validation cohort; **E** Multivariable ROC curves in the training cohort; **F** Multivariable ROC curves in the validation cohort

To further evaluate the role of the ARLnc signature in predicting prognosis, classical clinicopathological features and risk scores were include in the prognosis-related analysis. Univariate Cox regression showed that age, T stage, N stage, and risk score based on the four ARLncs signature were closely associated with survival in the training cohort and validation cohort (Fig. 3A, C). Additionally, the multivariate Cox regression results demonstrated that the prognostic independence of the risk score was demonstrated for OS in the training cohort (Hazard ratio HR = 1.102, CI = 1.053–1.153, *p* < 0.001) and validation cohort (HR = 1.097, CI = 1.054–1.141, *p* < 0.001) (Fig. 3B, D). The aforementioned features, including age (HR = 1.027, CI = 1.007–1.047, *p* = 0.007) and stage (HR = 1.517, CI = 1.066–2.157, *p* = 0.02), were both independent prognostic indicators of STAD in the validation cohort (Table 3). Furthermore, the accuracy of the ARLnc signature was evaluated with ROC analysis. The AUC of the ARLnc signature were 0.729 which was significantly higher than that of age (AUC = 0.622), gender (AUC = 0.584), stage (AUC = 0.607), T stage (AUC = 0.601) and N stage (AUC = 0.552), (Fig. 3E). In the validation cohort, the AUC of ARLnc signature was 0.634 which was higher than that of age (AUC = 0.605), gender (AUC = 0.499), stage (AUC = 0.589), T stage (AUC = 0.556), and N stage (AUC = 0.552) (Fig. 3F). These results indicated that the ARLnc signature better predicted the prognosis of STAD compared to traditional clinical factors.

**Table 3:** Univariate and multivariate Cox regression analysis of overall survival

Variables	Univariate analysis			Mulivariate analysis		
	HR	95%CI of HR	*p* value	HR	95%CI of HR	*p* value
Training cohort						
Age	1.036	1.005–1.068	0.021	1.051	1.017–1.087	**0.002**
Gender	1.503	0.811–2.783	0.195	1.446	0.764–2.737	0.256
Stage	1.682	1.208–2.342	0.002	1.6228	0.971–2.710	0.064
T	1.489	1.046–2.120	0.027	1.295	0.825–2.031	0.260
N	1.369	1.078–1.739	0.009	1.095	0.796–1.506	0.574
Riskscore	1.091	1.045–1.139	6.77E-05	1.102	1.053–1.153	**2.17E-05**
Validation cohort						
Age	1.020	1.001–1.039	0.036	1.027	1.007–1.047	**0.007**
Gender	1.348	0.898–2.023	0.148	1.248	0.825–1.886	0.393
Stage	1.502	1.203–1.876	0.003	1.517	1.066–2.157	**0.020**
T	1.295	1.025–1.636	0.030	1.063	0.781–1.445	0.695
N	1.270	1.075–1.501	0.004	1.029	0.824–1.286	0.796
Riskscore	1.093	1.053–1.136	3.16E-06	1.096	1.054–1.141	**4.64E-06**

*HR* Hazard ratio, *95%CI* Upper and lower limits for the hazard ratio

### Analysis of the angiogenesis status in different risk populations

**Fig. 4:**
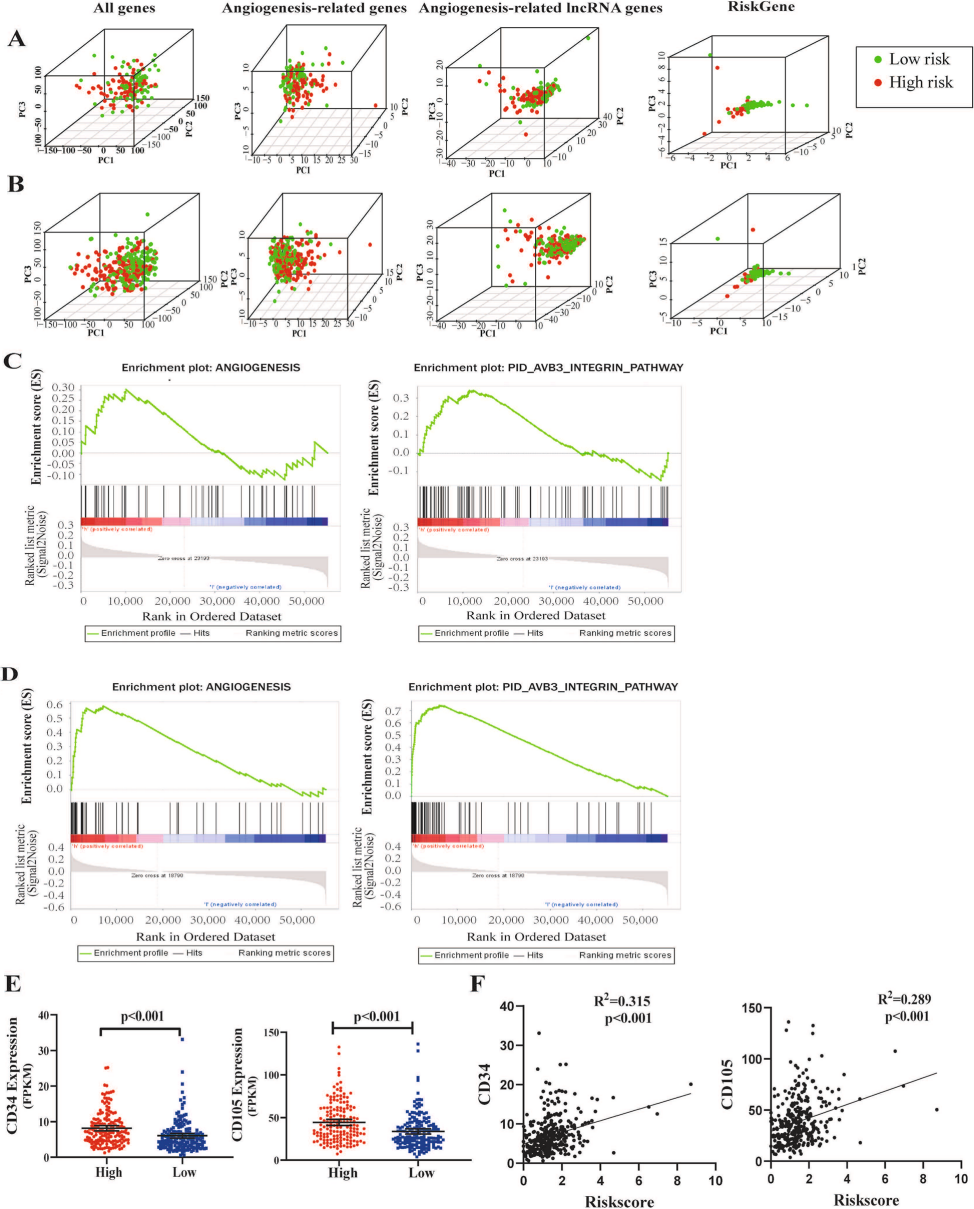
The high- and low-risk groups showed different angiogenesis state. **A**. PCA between high- and low-risk groups based on the all genes, angiogenesis-related genes, angiogenesis-related lncRNAs, and risk genes expression profiles in the training cohort; **B**. PCA between high- and low-risk groups on the all genes, angiogenesis-related genes, angiogenesis-related lncRNAs, and risk genes expression profiles in the validation cohort; Gene set enrichment analysis in the training (**C**) and validation cohort (**D**); **E** Gene expression levels between the high- and low-risk groups for CD34 and CD105, respectively; **F** The relationship between the risk score and angiogenesis-related markers (CD34 and CD105) in the validation cohort were analyzed by Pearson correlation

PCA was performed to detect the different distribution patterns between the low-risk group and the high-risk group based on all genes, angiogenesis gene set, lncRNA gene sets, and ARLnc gene sets in the training cohort (Fig. 4A) and validation cohort (Fig. 4B). The results showed that by using four ARLncs, STAD patients could be divided into two parts, and the angiogenesis status of STAD patients in the high-risk group differed from those in the low-risk group. The GSEA results further demonstrated that the expression of ARLnc genes in the high-risk group was enriched in angiogenesis-related responses and processes (NES = 1.90, FDR = 0.002) as well as integrin-related responses and processes (NES = 2.16, FDR = 0.000) in the training cohort (Fig. 4C). These results were further validated in the validation cohort (Fig. 4D). In addition, we analyzed the differential expression of CD34 and CD105, two significant markers of angiogenesis [13, 14], between the high- and low-risk groups in the validation cohort. As shown in Fig. 4E, the expression of CD34 was significantly increased in the high-risk group compared to that in the low-risk group (8.16 ± 4.68 vs. 6.05 ± 4.30, *p* < 0.001). Similar results were observed for CD105 (44.31 ± 23.31 vs. 33.65 ± 20.11, *p* < 0.001). To further characterize the angiogenesis status in tumor tissue, the relationship between the risk score and angiogenesis-related markers (CD34 and CD105) was determined utilizing Pearson correlation. Consistently, the angiogenesis risk score was positively correlated with the expression of CD34 (*R*^2^ = 0.315, *p* < 0.001) and CD105 (*R*^2^ = 0.289, *p* < 0.001) (Fig. 4F).

### Establishment and evaluation of the ARLnc clinical nomogram

**Fig. 5:**
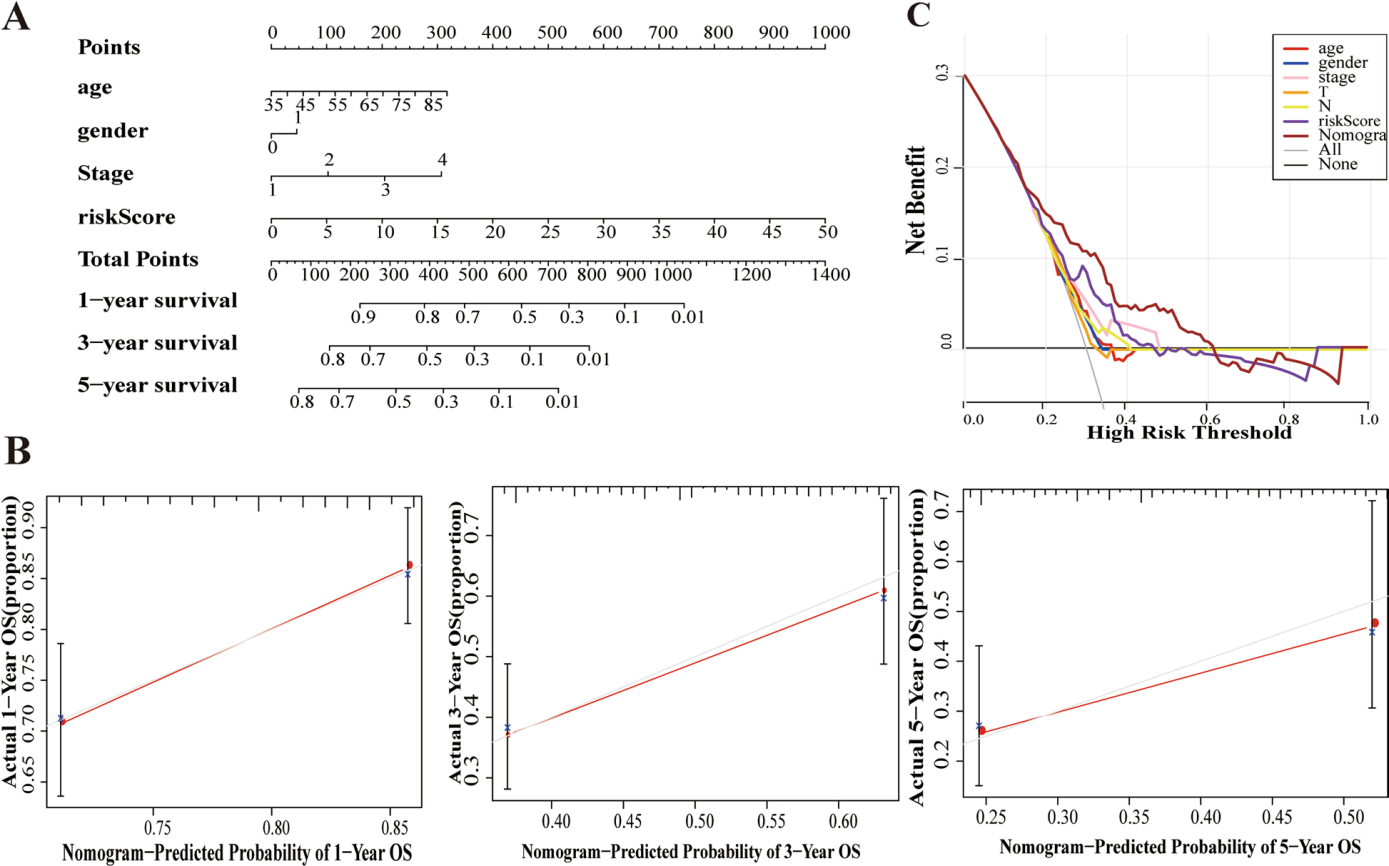
Construction and evaluation of nomogram for prognosis prediction of STAD patients. **A** The construction of nomogram combining four ARLnc signature with the clinical factors for indicting the OS of STAD patients; **B** The nomogram calibration curve to evaluate the prediction of 1-, 3-, and 5-year OS in STAD patients; **C** The decision curve analysis (DCA) for the nomogram. The net benefit was plotted versus the threshold probability. The brown line stood for the nomogram

The nomogram was established according to the results of multivariate Cox regression including age, gender, tumor stage, and risk score in the training cohort. This nomogram was used to quantitatively predict the prognosis of patients for 1-, 3- and 5-year survival (Fig. 5A). The concordance index (C-index) was calculated to assess the performance of prognostic nomogram discrimination. After bootstrapping with 1000 resampling as an internal validation, the C-index of the OS nomogram was 0.725 and 0.653 in the training and validation cohorts, respectively. Furthermore, the calibration curve showed that the prediction probabilities of the 1-, 3- and 5-year survival improved as the correction line became closer to the diagonal line (Fig. 5B). We further estimated the reliability of the nomogram by the DCA curve (Fig. 5C). The DCA curves confirmed that the nomogram had a better net benefit at different threshold probabilities, such as single tumor stage and risk score. Taken together, these findings indicated that the nomogram showed excellent performance and reproducibility for OS prediction. Collectively, our results showed that the prognostic model has better prediction accuracy that closely approximates the actual probabilities.

### Functional characteristics of the ARLnc signature

**Fig. 6:**
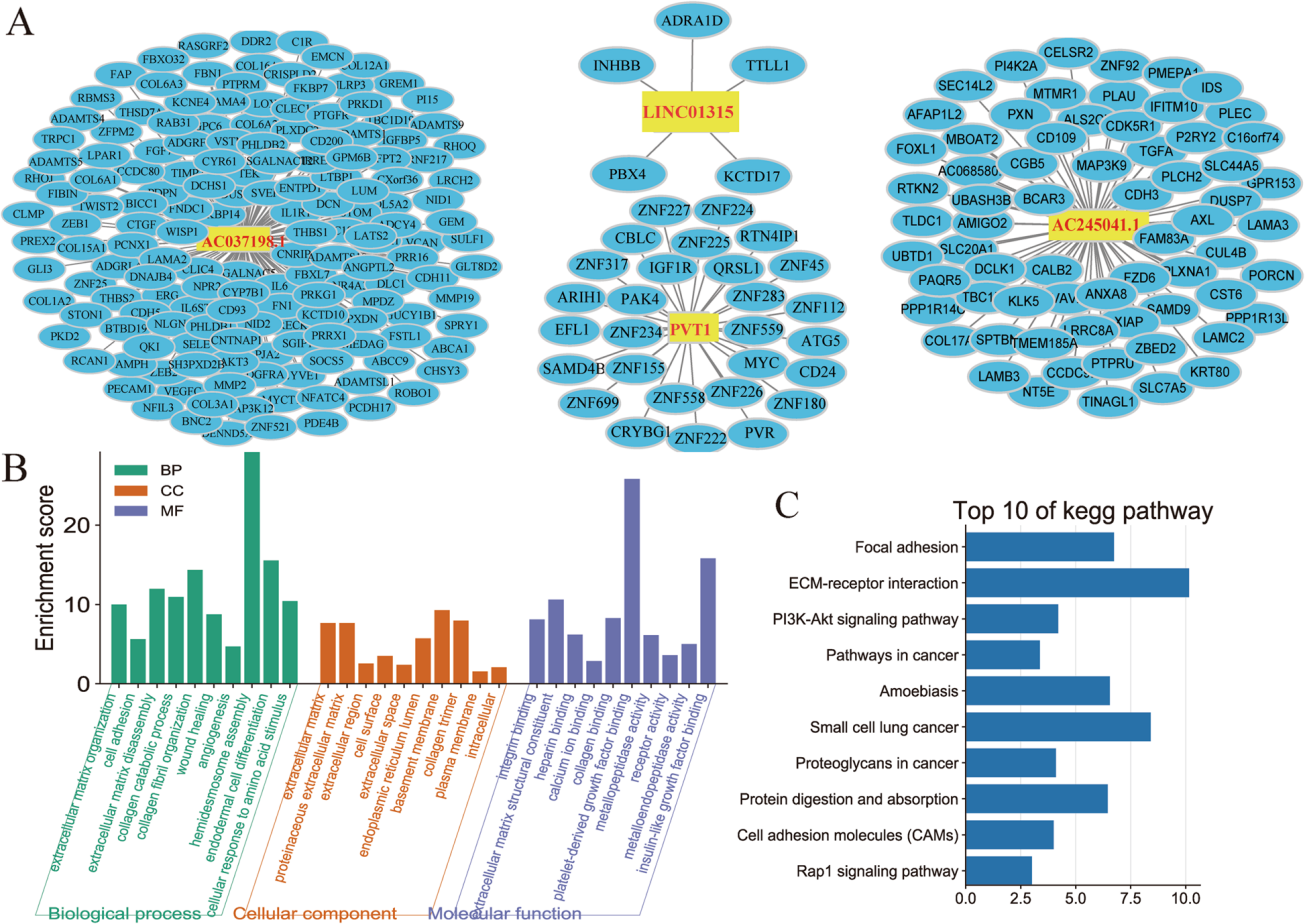
Potential functions of the four lncRNAs coexpressed mRNA. **A** lncRNAs and coexpressed mRNA network constructed using Cytoscape; **B** GO analysis on the biological processes (BP), cellular compoments (CC), and molecular functions (MF); **C** KEGG analysis on the enrichment pathway of coexpressed mRNA. The top 10 with *p* < 0.05 were selected for plotting

The following functional enrichment analysis was conducted to annotate the potential function of the ARLnc signature in STAD tumorigenesis and angiogenesis. After conditional screening, 262 coexpressed mRNAs positively correlated with the levels of at least one of the four ARLncs (Pearson correlation coefficient > 0.45, and FDR < 0.01), and a visualized ARLnc-mRNA network was established using Cytoscape (Fig. 6A). GO analysis revealed that these coexpressed mRNAs were mainly involved in several biological processes, such as angiogenesis, cell adhesion, wound healing, and extracellular matrix organization. (Fig. 6B). KEGG pathway analysis indicated that the top three significant enrichment pathways were focal adhesion, ECM receptor interaction, and PI3K-Akt signaling pathway (Fig. 6C), which are also closely associated with angiogenesis.

### The expression of four ARLncs associated with OS of STAD

**Fig. 7:**
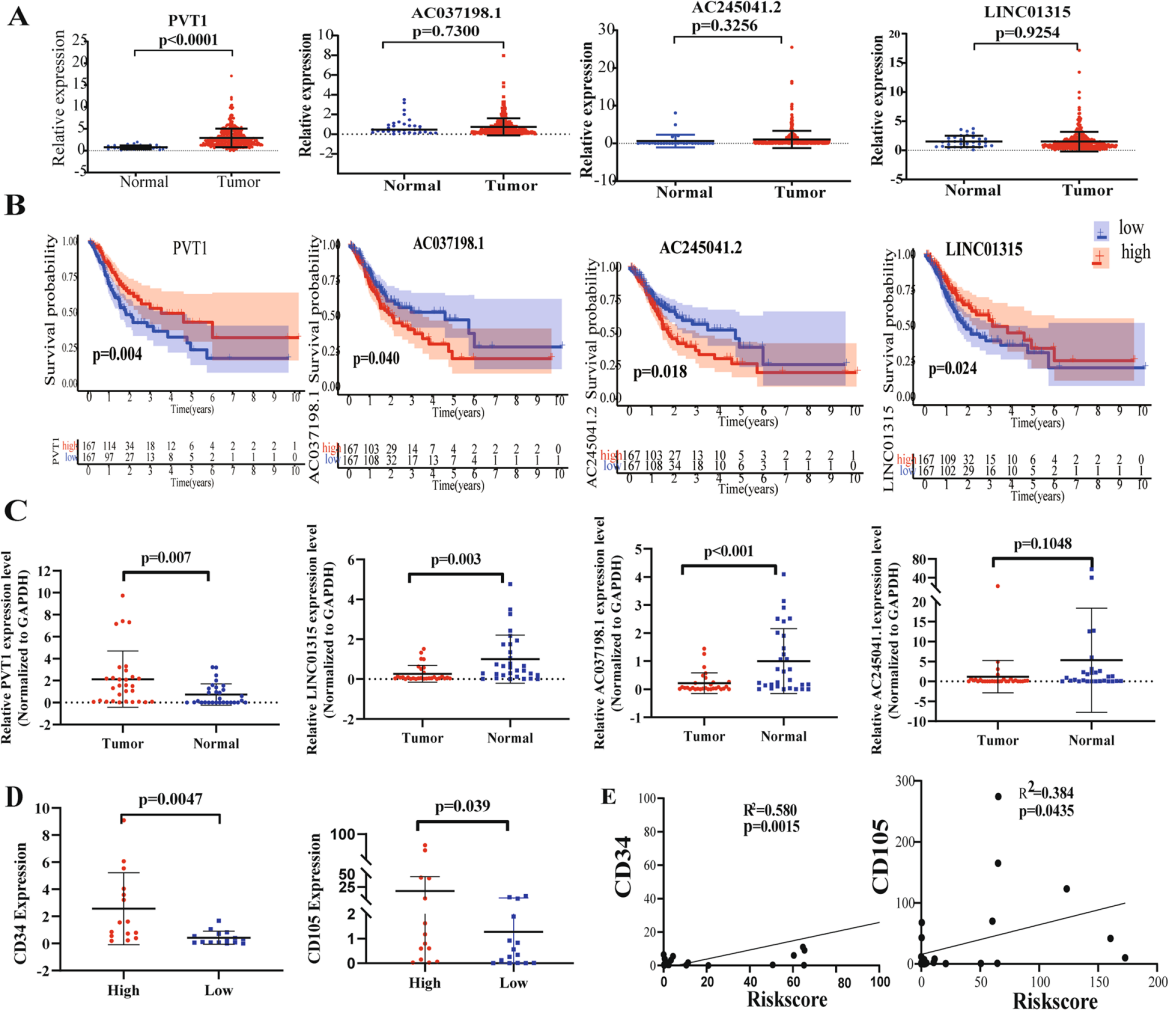
The expression of four ARLncs and correlation with the survival of STAD patients. **A** Differential expression of each lncRNA between STAD tumor tissues and normal tissues; **B** Kaplan-Meier survival curves of each lncRNA based on TCGA data for the probability of survival in STAD patients; **C** Gene expression levels between 30 STAD tumor tissues and paired adjacent normal tissues for LINC01315, AC245041.1, PVT1, and AC037198.1, respectively; **D** The relationship between the risk score and angiogenesis-related markers (CD34 and CD105) in 30 STAD samples were analyzed by Pearson correlation

The expression of each ARLnc was compared between STAD tumors (*n* = 375) and normal tissues (*n* = 32) in TCGA. There were no significant differences in the expression of LINC01315 (*p* = 0.9254), AC245041.2 (*p* = 0.3256), or AC037198.1 (*p* = 0.7300) between tumor and normal tissues. However, the expression of PVT1 was significantly upregulated in tumors compared to normal tissues (*p* < 0.001) (Fig. 7A).

According to the median expression level of the four lncRNAs, the difference in survival time between the two groups was analyzed by Kaplan–Meier survival analysis As shown in Fig. 7B, the expression of the four ARLncs was significantly associated with the survival of STAD patients (*p* < 0.05). In detail, the patients who highly expressed PVT1 and LINC01315 had longer OS than those who expressed lower levels (*p* = 0.004 and *p* = 0.024, respectively). In contrast, the patients who expressed high levels of AC037198.1 and AC245041.2 had shorter OS than those with lower levesl (*p* = 0.040 and *p* = 0.018, respectively).

### Validation based on clinical samples and correlation with angiogenesis

A clinical STAD cohort of 30 patients was established to validate the relationship between ARLnc and tumor angiogenesis. Relative expression of the four ARLncs in tumors and paired adjacent normal tissues was analyzed by RT–qPCR. The results showed that the expression of PVT1 in our cohort was significantly increased in the tumor tissues compared to the adjacent normal tissues (*p* < 0.001) (Fig. 7C). In addition, LINC01315 and AC037198.1 were expressed at lower levels in tumor tissues than in adjacent normal tissues (*p* < 0.05). The risk score was calculated according to the previous formula of the signature, and we further compared the expression of CD34 and CD105 between patients with high- and low- risk scores. The results confirmed that compared to the low-risk group, CD34 and CD105 levels were both significantly increased in the high-risk group (2.56 ± 2.65 vs. 0.42 ± 0.47, *p* = 0.0047; 17.04 ± 28.09 vs. 1.27 ± 2.08, *p* = 0.039, respectively) (Fig. 7D). Consistent with TCGA datasets, correlation analysis showed that there was a positive correlation between risk score and CD34 expression (*R*^2^ = 0.580, *p* = 0.0015) as well as between risks core and CD105 expression (*R*^2^ = 0.384, *p* = 0.0435) (Fig. 7E), thereby confirming the relationship between the signature and angiogenesis status.

## Discussion

LncRNAs are not only important regulators of cancer hallmarks but also key players in the pathogenesis of angiogenesis by regulating gene expression [15, 16]. Although these seminal studies demonstrated the contribution of lncRNAs throughout angiogenesis and their correlation with the prognosis of patients in small cohorts, panels of lncRNA biomarkers in conjunction with the status of the angiogenesis process may be better optimized than single lncRNAs.

In the present study, we first systematically analyzed the prognostic prediction accuracy of ARLncs in STAD. We established an angiogenesis-related prognostic signature based on the expression of four lncRNAs (LINC01315, AC245041.1, PVT1, and AC037198.1). Patients in the high-risk group had shorter OS than those in the low-risk group. It is worth mentioning that, in addition to angiogenesis gene database (Angiogenesis M14493), another integrin pathway-related gene database (INTegrin pathway M160) was investigated in the context of a more comprehensive strategy to define angiogenesis-related genes. Integrin is regarded as an bridge between vascular endothelial cells and the ECM [17], and positive integrin expression indicates poor prognosis of gastric cancer [18], suggesting an important role in the angiogenesis process.

Previous studies have shown that PVT1 is upregulated in poorly differentiated and advanced gastric cancer patients, and high- PVT1 levels predict shorter survival times, suggesting its potential diagnostic and prognostic value [19, 20]. In the present study, we revealed that the expression of PVT1 in STAD was significantly higher in tumor tissues than in adjacent normal tissues. However, further analysis with TCGA datasets showed that patients with high expression of PVT1 survived longer. This inconsistency between the expression level and OS indicated that PVT1 is not suitable as a single marker to predict prognosis. LINC01315 has been reported to be upregulated in nasopharyngeal carcinoma [21] and colorectal carcinoma (CRC), and it may be a prognostic biomarker in triple-negative breast cancer (TNBC) [22]. However, downregulation of LINC01315 has been found in oral squamous cell carcinoma (OSCC) [23]. Of note, LINC01315 was downregulated in our tumor samples, but no difference in expression was identified when analyzing datasets from TCGA. These contradictory findings highlighted the complexity of lncRNAs, which may be due to the influence of different tumor microenvironments or limited sample sizes, indicating that the development of improved strategies for deciphering their functions is needed. In addition, we found for the first time that AC245041.1 and AC037198.1, two unknown lncRNAs, are involved in the angiogenesis of STAD but their roles and molecular mechanisms in tumors have not been reported, thus requiring further studies.

The Nomogram is applicable in medical research and clinical practice due to their intuitionistic and easy to understand characteristics [24]. Our ARLnc signature-based nomogram relies on routinely available variables, including age, gender, tumor stage, and risk score. Moreover, calibration plots showed that the actual and predicted 1-, 3-, and 5-year survival rates based on our nomogram were better predictors, as indicated by the close proximity of the correction line to the diagonal line. The DCA curves further confirmed that the nomogram had a better net benefit at different threshold probabilities. Thus, the ARLnc prognostic signature accurately predicts the survival outcomes of STAD patients, allowing clinicians to easily estimate outcomes and make individual prognosis and therapy decisions.

Importantly, we further evaluated the expression of angiogenesis markers (CD34 and CD105) to characterize the angiogenesis status in 30 external STAD samples by the qRT–PCR. Our results revealed that the risk scores were associated with the expression levels of CD34 and CD105, indicating a close relationship between the prognostic model and angiogenesis status. Thus, the present study indicated that the evaluation of the risk model might also reflect the tumor angiogenesis status.

Compared to other prognostic signatures, our study mainly focused on lncRNAs correlated with angiogenesis status, identified prognosis-related ARLncs, and developed a risk model of prognosis in patients with STAD. Previous studies have investigated several prognostic models based on lncRNA expression involved in the process of ferroptosis [25], N6-methyladenosine modification [26], or *Helicobacter pylori* infection [27], which induce and activate the protumor signaling pathway of gastric cancer. Thus, our risk signatures had dual roles as it predicted the prognosis of STAD and contributed to the selection of proper cohorts who may benefit from antiangiogenesis treatment. However, there were several limitations in this study. First, given the heterogeneity, there are several tissue subtypes in gastric cancer (including microsatellite unstable (MSI), Epstein-Barr virus (EBV) positive, genomically stable (GS), and chromosomal instability (CIN) [28]), which require leveraging more retrospective cohorts including various subtypes. Additionally, while the samples in TCGA dataset were derived mainly from Western countries, further studies are required to investigate prevalent populations of gastric cancer in Eastern countries where lower proportions have signet ring histology and proximal stomach involvement [29]. Importantly, overcoming these limitations would require delineation of the molecular mechanisms of lncRNAs involved in angiogenesis.

## Conclusion

In summary, the present study constructed the ARLnc prognostic signature related to the survival of STAD patients, and the predictive efficacy of the signature was verified as an independent prognostic factor. These results provided new insights into the role of angiogenesis-related lncRNAs in gastric cancer, which will be helpful for prognosis and treatment.

## Supplementary Information

**Additional file 1 : Table S1.** The angiogenesis-related genes extracted from the Molecular Signatures Database v4.0. **Table S2.** A total of 329 lncRNAs were defined as ARLnc. **Table S3.** The sequences of primers utilized in this study.

## Data Availability

The data of this study are from The Cancer Genome Atlas (http://portal.gdc.cancer.gov/) and the Molecular Signatures Database v7.4 (Angiogenesis M14493, INTegrin pathway M160, http://www.gsea-msigdb.org/gsea/msigdb/).

## Abbreviations

TCGA: The cancer Genome Atlas
LncRNA: Long non-coding RNA
ARLnc: Angiogenesis-related long non-coding RNA
ARGs: Angiogenesis-related genes
OS: Overall survival
HR: Hazard ratio
STAD: Stomach adenocarcinoma
GSEA: Gene set enrichment analysis
PCA: Principal components analysis
GO: Gene Ontology
NES: Normalized enrichment score
FDR: False discovery rate
