# Supplementary material for: Angiogenesis-related lncRNAs predict the prognosis signature of stomach adenocarcinoma
Source: BMC Cancer. 2021 Dec 7;21:1312. doi: 10.1186/s12885-021-08987-y (PMC8653638; doi:10.1186/s12885-021-08987-y)
Supplement: Supplementary file 1 — Additional file 1 : Table S1. The angiogenesis-related genes extracted from the Molecular Signatures Database v4.0. Table S2. A total of 329 lncRNAs were defined as ARLnc. Table S3. The sequences of primers utilized in this study. [file 12885_2021_8987_MOESM1_ESM.zip › Table S3.docx]

Table S3 The sequences of primers utilized in this study

|  |  |  |
| --- | --- | --- |
| PVT1 | Forword (5’-3’) | CCTGTGACCTGTGGAGACAC |
|  | Reverse(5’-3’) | GCCATCTTGAGGGGCATCTT |
| LINC01315 | Forword (5’-3’) | GTACAGCTCCTGGCAGTGTG |
|  | Reverse(5’-3’) | CTGAAAACCCCAGCTACCCA |
| AC245041.1 | Forword (5’-3’) | AGGCAGACAACCAACTAGGC |
|  | Reverse(5’-3’) | TTGCAGACCTCCTATGAGCC |
| AC037198.1 | Forword (5’-3’) | TAGGCATTTCCAGTCTCA |
|  | Reverse(5’-3’) | AGCCAGAACCAACAACAG |
| GAPDH | Forword (5’-3’) | GGACCTGACCTGCCGTCTAG |
|  | Reverse(5’-3’) | GTAGCCCAGGATGCCCTTGA |
